# A Case of Lung Cancer with Brain Metastasis following Late-Onset Bipolar Disorder

**DOI:** 10.1155/2021/8880539

**Published:** 2021-04-01

**Authors:** Shin-Heng Shen, Shwu-Hua Lee

**Affiliations:** ^1^Department of Psychiatry, Chang Gung Memorial Hospital at Linkou, 333 Taoyuan, Taiwan; ^2^School of Medicine, Chang Gung University, 333 Taoyuan, Taiwan

## Abstract

**Objective:**

To describe a case of lung cancer with brain metastasis in a patient who developed new late-onset bipolar disorder 2 years previously.

**Background:**

The typical onset age of bipolar disorder is approximately 20, and the first episode is usually a depressive episode. It is still not clear which age-specific factors contribute to the underlying risk.

**Materials and Methods:**

A 65-year-old male patient presented with a new-onset manic episode characterized by labile mood, impulsivity, decreased need for sleep, and grandiosity. He was diagnosed with late-onset bipolar disorder after excluding other possible physiological conditions. He was hospitalized in the acute psychiatric ward, and a combination of mood stabilizers and antipsychotics was prescribed. His mental condition improved, and he remained stable for 2 years. However, he experienced abrupt cognitive decline for 2 months and was referred to the emergency room for physiological examination.

**Results:**

The patient was diagnosed with lung cancer with brain metastasis by brain magnetic resonance imaging and whole-body positron emission tomography.

**Conclusion:**

In geriatric patients, who are at high risk of multiple medical conditions, excluding secondary causes of bipolar disorder is important.

## 1. Introduction

Bipolar disorder manifests as manic/hypomanic episodes and depressive episodes. Typically, the onset of bipolar disorder occurs at approximately the age of 20, and the first episode is usually a depressive episode [1]. Among all patients with bipolar disorder, 25% are older than 60 years [2]. Approximately 10% of late-onset bipolar disorder patients develop new-onset mania later in life, often associated with vascular changes or other brain disorders [3, 4]. If a patient who does not have a previous psychiatric history presents with new-onset manic-like symptoms, it is the psychiatrist's responsibility to distinguish true bipolar disorder from nonpsychiatric causes. According to the definition in the *Diagnostic and Statistical Manual of Mental Disorders* fifth edition (DSM-5), a manic episode should not be attributable to the physiological effects of a substance or to another medical condition [5]. In other words, if a patient has atypical onset or an atypical disease course, psychiatrists need to exclude secondary causes. In particular, in geriatric patients, who have high rates of multiple medical conditions and polypharmacy, complete physical examination is necessary [6].

In an acute manic episode, a medical condition or comorbidity may occur independently of mania but could also be the cause of mania (secondary mania) [4]. The causal relationship is hard to establish; however, appropriate psychiatric and physiological workups and monitoring can be provided. Here, we describe a geriatric patient with new-onset mania who initially did not have any known medical condition but for whom lung cancer with brain metastasis was discovered 2 years after the initial onset of bipolar disorder.

## 2. Case Report

Mr. F, a 65-year-old male, was brought to the psychiatric outpatient clinic by his family who reported labile mood, impulsivity, decreased need for sleep, grandiosity, talkativeness, and increased goal-directed behaviors for 3 weeks. Several instances of physical aggression toward family members and self-harm were also reported. He also reported auditory hallucinations without visual hallucinations. A trigger event occurred one month previously, when his affairs were discovered by his family. He initially became depressed and anxious and then experienced mania, as mentioned above, a week later. The patient was oriented and communicable at the clinic despite his irritability. Late-onset bipolar I disorder was diagnosed, and a combination of valproic acid 500 mg/day and olanzapine 10 mg/day was prescribed for acute mania. The subsequent laboratory examination revealed balanced electrolytes, normal thyroid function, and negative syphilis rapid plasma reagin (RPR). Electroencephalography (EEG) showed mild diffuse cortical dysfunction.

The patient's condition improved gradually within the first week of psychiatric treatment. However, he became more agitated and paranoid thereafter, with aggravated auditory hallucinations and persecutory delusions. These symptoms did not improve despite titrating valproic acid to 1000 mg/day and switching from olanzapine to paliperidone 9 mg/day. He was hospitalized in the acute psychiatric ward, and his blood tests and chest X-ray were unremarkable.

In the first 2 weeks of hospitalization, the patient remained agitated and paranoid, and he was frequently placed in a seclusion and protection room (SPR) due to concerns about safety and maintaining his sleep schedule. With the medication adjustment, the patient's clinical condition improved gradually. He was discharged after 6 weeks of hospitalization; the discharge regimen was valproic acid 500 mg/day, aripiprazole 20 mg/day, and quetiapine 150 mg/day. Psychological tests before discharge were performed; the cognitive abilities screening instrument-2.0 (CASI-2.0) score was 82.5/100, and the clinical dementia rating scale (CDR) score was 0.5.

In the following 8 months, his mood remained stable and no psychosis was observed; therefore, the medication was gradually tapered to valproic acid 500 mg/day and quetiapine 50 mg/day. We performed brain magnetic resonance imaging (MRI) to exclude other physiological causes of late-onset bipolar disorder; the findings showed only old, small insults and atrophy. However, after remaining stable for another year, the patient developed acute cognitive function decline and an unsteady gait for 2 months. Laboratory blood tests were unremarkable, while cerebral positron emission tomography (PET) showed decreased [^18^F]2-fluoro-2-deoxy-D-glucose (FDG) uptake in the cortex regions, compatible with typical Alzheimer's disease. Psychological tests revealed a CASI-2.0 score of 73.4/100 and a CDR score of 0.5, so rivastigmine 9 mg was given. Despite the prescription of antidementia drugs, the patient experienced exacerbations of disorientation, paranoia, delusions involving theft, delusions involving jealousy, visual hallucination, irritability, negative thoughts, insecure feelings, and inappropriate behaviors. These symptoms were considered to be attributed to behavioral and psychological symptoms of dementia (BPSD).

**Figure 1:**
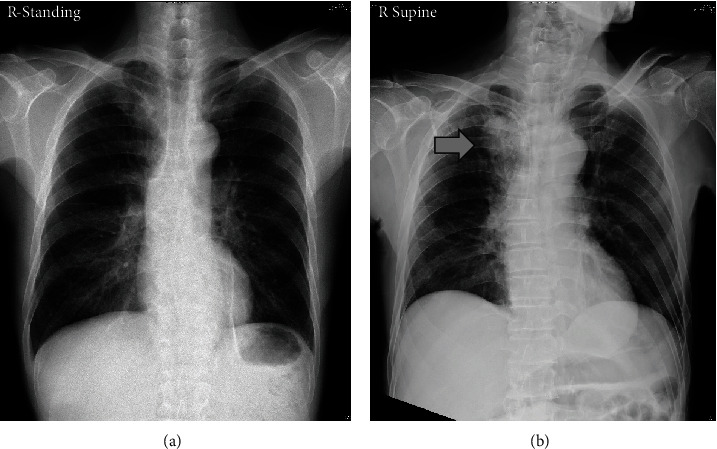
Unremarkable chest X-ray at (a) mania onset and (b) 2 years later, with a mass (arrow) in the right upper lobe of the lung.

**Figure 2:**
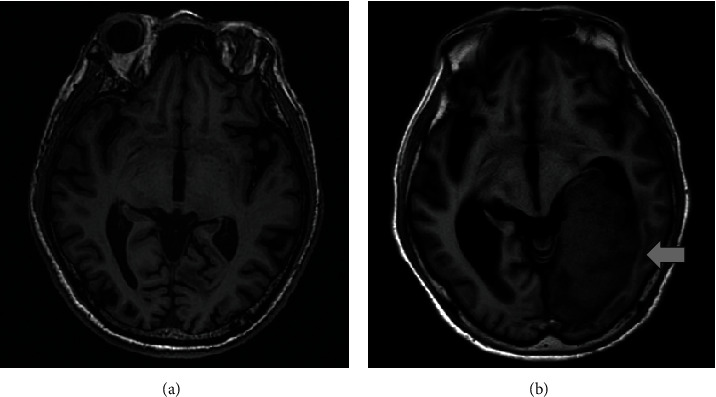
Brain MRI (a) 10 months after and (b) 2 years after mania onset, with a brain mass at 2 years (arrow).

One month later, a neuropsychological assessment was performed because of the presentation of severe clinical dementia, with a CDR score of 2 (Table 1). His rapidly declining cognitive function was alarming, but there were no remarkable neurologic deficits or physiological abnormalities at that time. Three months later, his family reported progressive left side extremity weakness and frequent choking for a week, so he was referred to the emergency room (ER) by the outpatient clinic. Laboratory blood tests were still unremarkable, but a chest X-ray showed a mass in the right upper lobe of the lung (Figure 1). Brain computed tomography (CT) and MRI revealed a mass lesion in the left occipito-parietotemporal lobe, sized 8.4 cm × 4.4 cm × 6.0 cm, with a midline shift to the right (Figure 2). Malignant origin was favored, and neurosurgery specialists were consulted for evaluation.

Given the concern about the effect of this large tumor and the rapid progression of neurologic deficits, the patient underwent emergency tumor removal surgery. The pathological report showed metastatic adenocarcinoma of pulmonary origin. Whole-body CT with a contrast agent revealed a necrotic lung mass in the right upper lobe and a solid lung nodule in the left upper lobe. FDG PET-CT confirmed right upper lung cancer with lung-to-lung and distant brain metastasis. The patient was then transferred to a pulmonary oncologist for chemotherapy.

The patient did not take any psychiatric medication after surgery; however, valproic acid 1000 mg/day was prescribed by the neurosurgery specialist for postbrain surgery seizure prophylaxis.

## 3. Discussion

Evidence has shown that compared to early-onset bipolar disorders, late-onset disorders are more frequently associated with atypical features, organic brain abnormalities, vascular injuries, and neurological illnesses [7–9]. Therefore, psychiatrists need to exclude any possible physiological causes (secondary mania, delirium, and dementia) of a late-onset manic episode [10]. The diagnostic workup for late-onset mania includes history taking (medical, psychiatric, medication, and substance), cognitive function assessment, laboratory examinations (blood count, electrolytes, renal and liver function, thyroid function, vitamin B, folic acid, and serum levels of medications), brain imaging, and EEG [4].

In patients with brain tumors, psychiatric symptoms, including mood-related or psychotic symptoms, cognitive problems, and personality changes, are common [11]. The frequency of brain metastasis from lung cancer might be as high as 48% [12]. Moreover, lung cancer itself could cause neuropsychiatric syndromes, most commonly behavioral and cognitive changes, despite the absence of brain lesions [13]. These medical condition-related psychiatric symptoms, especially irritability, agitation, unusual behaviors, and sleep problems, may be similar to those of primary psychiatric disorders and often present challenges in making a differential diagnosis.

In this case, the patient presented with new-onset mania at age 65 and was diagnosed with bipolar disorder. We performed detailed examinations at the beginning of the manic episode, and all tests were negative for underlying medical conditions. Ten months later, brain MRI revealed only atrophy without any solid lesions. The above course made the diagnosis of late-onset bipolar disorder seem reasonable. However, one year later, he suddenly developed neurologic symptoms, and brain metastasis of pulmonary origin was found.

Previous studies have reported cases of new late-onset mania associated with lung cancer, with or without brain metastasis [14, 15]; in both studies, the patients had underlying lung cancer and developed subsequent manic symptoms. In our case, there were no notable lung or brain lesions initially. It is hard to determine whether the patient's mania was a consequence of lung/brain cancer or due to an independent condition. However, since many clinical symptoms of lung cancer occur late in the natural course of the disease, many patients are diagnosed at an advanced stage [16, 17]; therefore, it is possible that our patient had underlying lung lesions that were unable to be detected on the chest X-ray performed at the onset of acute mania. Since somatic health issues in patients with bipolar disorder are usually underrecognized and suboptimally treated [18], psychiatrists should give more attention to clinical multidisciplinary vigilance.

## 4. Conclusions

For patients who present with an unusual course or atypical features of acute mania, psychiatrists should complete possible and necessary examinations to exclude physiological or substance-related cases. In particular, in geriatric patients, who are at risk of having multiple medical conditions, excluding secondary causes of bipolar disorder is important not only at the beginning of an episode but also during the follow-up period and entire disease course.

## Figures and Tables

**Table 1:** Summary of neuropsychological assessment results during the 2 years of follow-up.

Months after mania onset	2	22	23
CASI-2.0 (total points)	82.5/100	73.4/100	—
Long-term memory	10/10	10/10	—
Short-term memory	10.5/12	6.4/12	—
Attention	8/8	8/8	—
Calculation	9/10	10/10	—
Orientation	12/18	10/18	—
Abstract thinking	11/12	9/12	—
Language	10/10	9/10	—
Visual construction	5/10	4/10	—
Category fluency	7/10	7/10	—
CDR	0.5	0.5	2
Memory	0.5	1	2
Orientation	0.5	1	2
Judgment-problem solving	0	0.5	1
Community affairs	0	0.5	2
Home hobbies	0	0	2
Personal care	0	0	1

The results were rated by clinical psychologists using the standardized Mandarin version of the cognitive abilities screening instrument-2.0 (CASI-2.0) and clinical dementia rating scale (CDR).

## Data Availability

Data are available from the medical chart and database of Chang Gung Memorial Hospital. Due to legal restrictions imposed by the government of Taiwan in relation to the “Personal Information Protection Act,” data cannot be made publicly available.
